# Impact of nutrition on inflammation, tauopathy, and behavioral outcomes from chronic traumatic encephalopathy

**DOI:** 10.1186/s12974-018-1312-4

**Published:** 2018-09-24

**Authors:** Jin Yu, Hong Zhu, Saeid Taheri, William Mondy, Stephen Perry, Mark S. Kindy

**Affiliations:** 1 Department of Pharmaceutical Sciences, College of Pharmacy, University of South Florida, 12901 Bruce B. Downs Blvd., MDC 30, Tampa, FL 33612 USA; 2 NutriFusion®, LLC, 10641 Airport Pulling Rd., Suite 31, Naples, FL 34109 USA; 3 Departments of Molecular Medicine, Molecular Pharmacology, Physiology and Pathology and Cell Biology, and Neurology, College of Medicine, University of South Florida, Tampa, FL USA; 4 James A. Haley VA Medical Center, Tampa, FL USA; 5 Shriners Hospital for Children, Tampa, FL USA

**Keywords:** Animal model, Chronic traumatic encephalopathy, Concussion, Pathophysiology, Repetitive, Diet, Inflammation, Neurodegeneration, Behavior

## Abstract

**Background:**

Repetitive mild traumatic brain injuries (rmTBI) are associated with cognitive deficits, inflammation, and stress-related events. We tested the effect of nutrient intake on the impact of rmTBI in an animal model of chronic traumatic encephalopathy (CTE) to study the pathophysiological mechanisms underlying this model. We used a between group design rmTBI closed head injuries in mice, compared to a control and nutrient-treated groups.

**Methods:**

Our model allows for controlled, repetitive closed head impacts to mice. Briefly, 24-week-old mice were divided into five groups: control, rmTBI, and rmTBI with nutrients (2% of NF-216, NF-316 and NF-416). rmTBI mice received four concussive impacts over 7 days. Mice were treated with NutriFusion diets for 2 months prior to the rmTBI and until euthanasia (6 months). Mice were then subsequently euthanized for macro- and micro-histopathologic analysis for various times up to 6 months after the last TBI received. Animals were examined behaviorally, and brain sections were immunostained for glial fibrillary acidic protein (GFAP) for astrocytes, iba-1 for activated microglia, and AT8 for phosphorylated tau protein.

**Results:**

Animals on nutrient diets showed attenuated behavioral changes. The brains from all mice lacked macroscopic tissue damage at all time points. The rmTBI resulted in a marked neuroinflammatory response, with persistent and widespread astrogliosis and microglial activation, as well as significantly elevated phospho-tau immunoreactivity to 6 months. Mice treated with diets had significantly reduced inflammation and phospho-tau staining.

**Conclusions:**

The neuropathological findings in the rmTBI mice showed histopathological hallmarks of CTE, including increased astrogliosis, microglial activation, and hyperphosphorylated tau protein accumulation, while mice treated with diets had attenuated disease process. These studies demonstrate that consumption of nutrient-rich diets reduced disease progression.

## Background

Mild traumatic brain injury (mTBI) is a result of concussive head traumas that are considered a growing issue, with millions of sports-, military-, and recreation-related concussions occurring each year [1, 2]. In the USA alone, over four million concussions occur each year, which is a considerable problem [3, 4]. Evidence from various studies on the physical properties, neuroimaging, neuropathology, and basic science experiments has determined that these concussive injuries and related subconcussive impacts have led to the development of both acute and chronic post-traumatic sequelae [5, 6]. Recently, the discovery of chronic traumatic encephalopathy (CTE) following “repetitive head injuries” has been seen in the majority of sports programs including football, soccer, hockey, and boxing, and is a serious problem [7–10].

Much of the information we have about CTE has been derived from data collected from autopsies and retrospective and population studies [11]. Because of the nature of the disorder, the incidence and prevalence are difficult to determine [12]. The risk factors associated with the development and progression of CTE are not well known, yet we understand that repetitive blunt force trauma to the head and body, blast impacts, and acceleration-deceleration influences can trigger the processes [13]. CTE has a myriad of clinical presentations that include impairments in cognition, behavior, and mood, and in some cases, chronic headache and motor and cerebellar dysfunction [14]. Behavioral changes such as irritability, judgment issues, increased risk-taking, and depression are characteristic and prominent early in the disease course. Unfortunately, the only way to diagnose the disease is through histological and immunohistochemical analyses that show the presence of hyperphosphorylated tau as multifocal or diffuse cortical and subcortical regions within the brain [15, 16]. In addition, the presence of inflammation during CTE is accompanied by the activation of astrocytes and microglial cells [17].

The mechanisms associated with the pathophysiological changes seen in CTE are still not well documented. Because of this, researchers have attempted to generate paradigms that best define the clinical evidence [18–20]. Various models of CTE have been developed including closed head repetitive mild traumatic brain injury (rmTBI) to mimic the pathological outcomes [21]. CTE may be a compilation of co-morbidities, normal or accelerated aging, or other factors that contribute to the symptomology [22]. Based on current data, following a TBI (or multiple TBIs), neurodegenerative conditions set in years afterwards, which is why accumulation of information takes a significant time to understand the potential mechanisms involved [23]. Over the years, experimental models that are representative of CTE and the neurological sequelae such as post-concussion syndrome (PCS), post-traumatic stress disorder (PTSD), and mild cognitive impairment (MCI) may be well represented by repetitive brain injury [24]. The behavioral patterns observed in CTE patients include cognitive deficits, increased risk-taking, depression-like behavior, and sleep disturbances [25, 26]. Therefore, rmTBI models result in the histopathological hallmarks of CTE, including increased astrogliosis, microglial activation, and phosphorylated tau immunoreactivity.

In the current study, our goal was to determine the influence of diets rich in vegetables and fruits on the outcomes associated with rmTBI or CTE. Mice were fed diets enriched in fruits and vegetables for 2 months and then subjected to rmTBI. The results demonstrated that diets high in phytonutrients were able to attenuate the “CTE-like” pathology provoked by the rmTBI. Behavioral changes, inflammation, and tau pathology were examined in mice chronically exposed to the diets. The results suggest that supplementation of mice with the enhanced diets limited the extent of the CTE, reduced inflammation, and altered pathways typical of CTE. These data suggest that these diets may be beneficial in altering the presentation of CTE seen in models of rmTBI and improve outcome.

## Methods

### Animal care and maintenance

All animals used in this study were treated in accordance with the National Institutes of Health Guidelines for the Care and Use of Laboratory Animals, and all procedures were performed under the approval of the Institutional Animal Care and Use Committee at the University of South Florida. Adult male, human Tau mice (hTau, Taconic, Hudson, NY) were purchased and housed with five mice per cage. Animals were 24 weeks of age at the start of the experiment and were maintained on a 12-h light/dark cycle (lights on at 7:00 a.m.). All animals were randomized to the various groups. Prior to TBI, animals were fed for 2 months a normal diet or a normal diet with ∼ 2% supplementation of the different materials NF-216 (GrandFusion – Fruit and Veggie #1 Blend), NF-316 (GrandFusion – Fruit #2 Blend), and NF-416 (GrandFusion – Vegetable #3 Blend) [27]. See Table 1 for composition of supplementation. In addition to the vitamins, through the isolation/extraction process, the phytonutrients in the fruits and vegetables are maintained and non-oxidized. Animals were gavaged with the supplements on a daily basis, once per day. GrandFusion supplements were prepared by NutriFusion, LLC (www.nutrifusion.com). Average food intake was 3.81 ± 0.08 g/day/mouse, and the average consumption of diets was 0.09 ± 0.006 g/day/mouse.

**Table 1:**
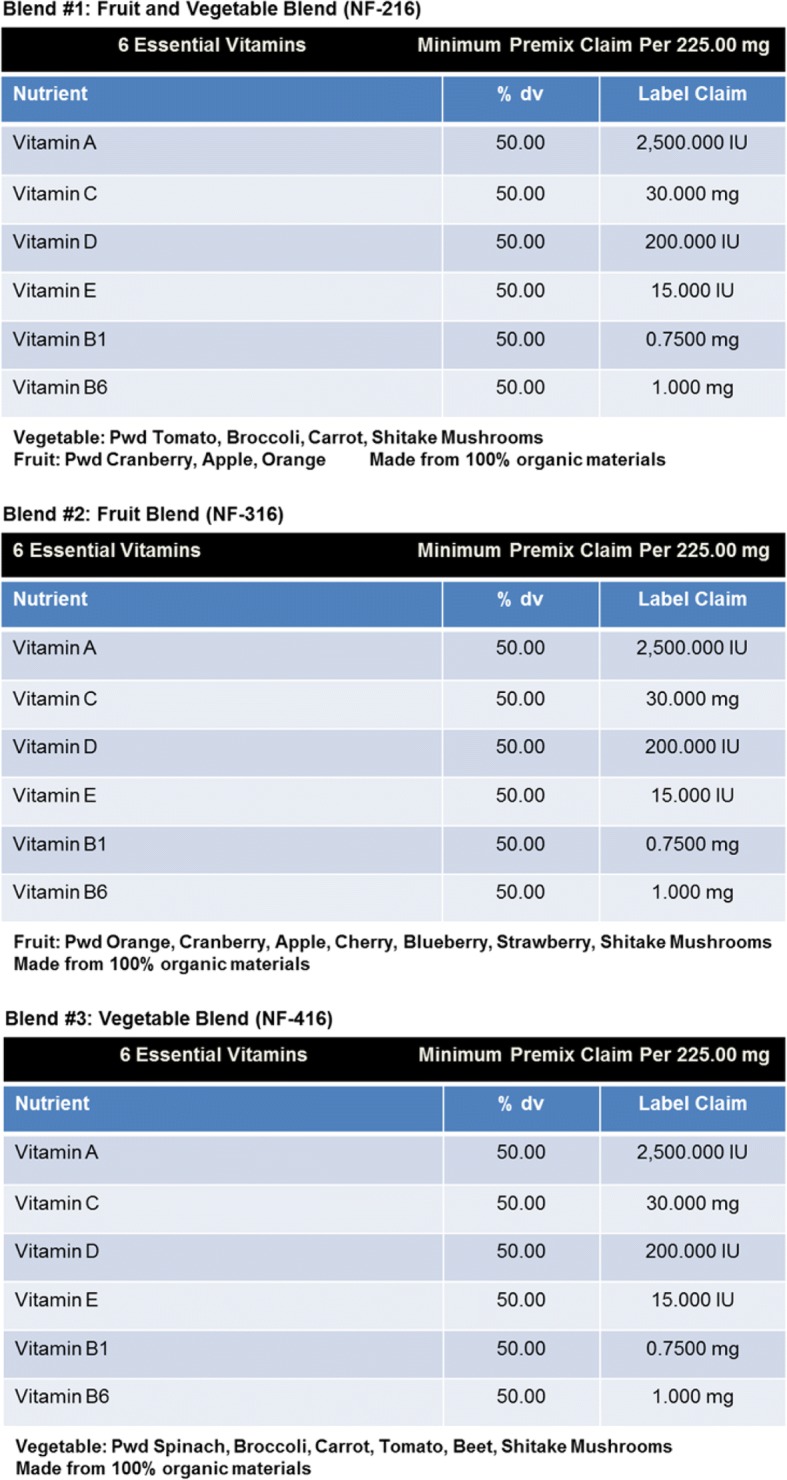
Composition of GrandFusion supplements

### TBI injury

The rmTBI mouse model was used to deliver a controlled, consistent injury to all animals [28]. Adult mice were anesthetized with ketamine (60–90 mg/kg) and xylazine (6–9 mg/kg) or isoflurane (5% induction, 1–2% maintenance). The degree of anesthesia was assessed by testing of interdigital pinch withdrawal reflex. Lacrilube ophthalmic ointment was applied to both eyes to prevent drying. For the repetitive closed head injury, following anesthesia, the mouse was placed in the pneumatic impactor device (Precision Systems and Instrumentation, Fairfax Station, VA) and was subjected to a closed head injury of 4 m/s (speed), 3.8 mm (depth), and 200 ms (dwell time). The mouse was returned to its home cage and monitored until it is awake. The procedure was repeated up to three times (four total injuries), spaced 2–3 days apart (i.e., M, W, F, M). Animals were returned to their home cages after recovery from anesthesia and monitored daily for any signs of discomfort or other abnormal behavior. See diagram for outline of behavioral testing.
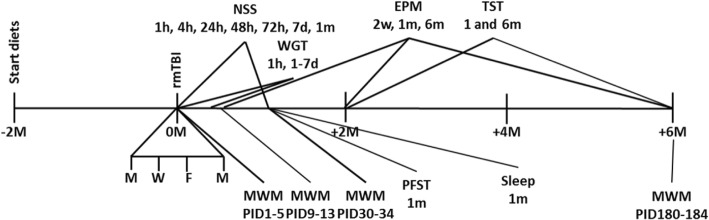


### Neurological Severity Score

To characterize the effects of the nutritional diet on repetitive mTBI/CTE in this model, a Neurological Severity Score (NSS) was used to evaluate the neurological impairment, compared to uninjured controls, as previously described [29]. The NSS is a composite clinical score consisting of 10 individual clinical parameters, including tasks on motor function, alertness, and general physiological behavior (Table 2). Mice (10 per group) were tested at 1, 4, 24, 48, and 72 h post-injury, as well as at 7-day and 1-month time points. Severity of injury was defined by the initial NSS measured at 1 h post-TBI and is a reliable predictor of late outcome [29].

**Table 2:** Neurological Severity Score

Task	Description	Score (success/failure)
Exit circle	Ability and initiative to exit a circle of 30 cm in diameter within 3 min	
Monoparesis/hemiparesis	Assess paresis of upper and/or lower limb	0/1
Straight walk/gait	Initiative and motor ability to walk straight	
Startle reflex	Innate reflex assessment; mouse should bounce in response to a loud hand clap	0/1
Seeking behavior	Physiological behavior as a sign of “interest” in the environment	
Beam balancing	Ability to balance on a beam of 7 mm in width for at least 10 s	0/1
Round stick balancing	Ability to balance on a round stick of 5 mm in diameter for at least 10 s	
Beam walk: 3 cm	Ability to cross a 30-cm-long beam: 3-cm-wide beam	0/1
Beam walk: 2 cm	Ability to cross a 30-cm-long beam (increased difficulty): 2-cm-wide beam	
Beam walk: 1 cm	Ability to cross a 30-cm-long beam (increased difficulty): 1-cm-wide beam	0/1
Total score		Of 10

Adapted from [29]. One point is awarded for the lack of a tested reflex or for the inability to perform the tasks outlined in the table, and no point for succeeding. A maximal Neurological Severity Score (NSS) of 10 points thus indicates severe neurological dysfunction, with failure of all tasks, and a normal healthy mouse would get a score of a zero on the tasks above. Roughly, NSS score: 1–4, mild TBI; 5–7, moderate TBI; 8–10, severe TBI

### Assessment of motor function

Vestibulomotor function was determined by a wire grip test (WGT) 1 h after TBI and on post-injury days 1–7 [30]. Mice (10 per group) were picked up by the tail and placed on a metal wire suspended between two upright bars 30 cm above a padded floor. The mice were assessed for the time and manner they could hold onto the wire and were recorded and scored on a scale of 0–5. Mice were tested three consecutive times at each of the indicated time points. The score reported is the average of these individual trials by individuals blinded to the treatments. A composite group score was then calculated as the mean of these scores at each time point and then used for analysis.

### Assessment of spatial learning and memory

Control and repetitive mTBI groups were tested in the Morris water maze (MWM) acutely (acquisition trials on post-injury days [PIDs] 1–5 and probe trial on PID 6), subacutely (acquisition trials on PIDs 9–13 and probe trial on PID 14), and chronically at 1 (acquisition trials on PIDs 30–34 and probe trial on PID 35) and 6 months (acquisition trials on PIDs 180–184 and probe trial on PID 185) after the final head impact. Mice had to locate an invisible platform submerged 5 mm below the water level in a circular pool (dimensions, 90 × 60 cm; temperature, 24 ± 3 °C), based on the spatial location of six strategic visual cues fixed at distinct positions around the pool. The water was made opaque by adding nontoxic, water-soluble tempera paint. Data were recorded with the help of video cameras (SMART video tracking system, San Diego Instruments, San Diego, CA).

For training days (acquisition phase), all mice (15 per group, per time point) were given a maximum test duration of 60 s to find the hidden platform. The latency to reach the platform was recorded by the video tracking system. Mice that failed to locate the platform within the time limit were guided to it and allowed to rest and orient themselves for 15 s. The acquisition phase testing was conducted over five consecutive days, with four trials on each day, with the goal of locating the submerged hidden platform from different starting points and orientations (north, south, east, and west). On day 6 of MWM testing, all animals were tested for visual acuity and swimming speed using a visible platform paradigm. None of the animals were excluded from further testing based on the visual acuity and motor evaluation tests. On day 6, all mice also underwent a probe trial (retention phase), where the platform was removed from the pool. Mice were given 30 s to swim, and time spent in the target quadrant (quadrant where the platform had been) versus the other quadrants was assessed as described previously.

### Assessment of anxiety-related and risk-taking behaviors

Anxiety-related and risk-taking behavior of the mice was evaluated using the elevated plus maze (EPM) test. Mice (15 per group, per time point) were evaluated at 2 weeks, 1 month, and 6 months from the last head impact. The EPM consisted of two opposing open arms (35 × 5 cm) and two closed arms (35 × 5 × 15 cm) that extended from a central platform (5 × 5 cm) elevated 60 cm above the floor. A small raised lip (0.5 cm) around the edges of the open arms prevents animals from slipping off. Mice were placed individually on the central platform facing an open arm, away from the examiner, and were allowed to freely explore the maze for 5 min under even overhead fluorescent lighting. The behavior of each mouse was monitored using a SMART video tracking system (San Diego Instruments, San Diego, CA). Time spent in the open and closed arms was determined, and each mouse was only tested once in the maze.

### Assessment of depression-like behavior

To determine the long-term effects of mTBI on depression-like behavior, mice (15 per group) were tested in the Porsolt forced swim test (PFST) and the tail suspension test (TST) at 1 month post-injury [31, 32].

### Porsolt forced swim test

Mice were placed in an open glass cylinder (diameter 12 cm, height 24 cm, and water level 16 cm) containing water at 23–25 °C. The time for the test was 6 min, with the first 2 min for habituation and the last 4 min used for analysis. Two different experimenters were blinded to the groups of mice evaluated for behavior, manually. A mouse was judged to be immobile when it remained floating in the water, making only those movements necessary to keep its head above the water surface.

### Tail suspension test

Briefly, mice were suspended by the tail to a bar elevated 40 cm above the surface of a table. The duration of the test was 6 min. Two different experimenters blinded to the groups of mice manually evaluated the behavior. The immobility time of the tail-suspended mice was measured and defined as the absence of limb movement. These tests were done at 2 and 6 months.

### Assessment of sleep behavior

Electroencephalography (EEG) and electromyography (EMG) data were acquired in mice at 1 month post-injury (15 per group) using implantable telemetry devices (Data Sciences International, ST. Paul, MN) and the Dataquest A.R.T. system (Data Sciences International). The transmitter was implanted intraperitoneally through a mid-line abdominal incision. EEG lead implantation was performed by insertion of leads in small burr holes overlying the cortex. EMG leads EMG electrodes were implanted into the neck muscle. The EEG electrodes were secured with dental cement, and the EMG electrodes were secured with sutures. Mice were allowed to recover and were individually housed in a sound-attenuated and ventilated chamber on a standard light/dark cycle, with food and water available ad libitum. After a 7-day acclimation and recovery period, the telemetry EEG/EMG devices were activated and continuous recordings were obtained for a total of 24 h (6 pm to 6 pm) [33]. EEG and EMG data were analyzed in 1-min epochs using Neuroscore software devices (Data Sciences International). Using both manual scoring and automated software, EEG/EMG recordings were broken down into active wake, non-rapid eye movement (NREM) sleep, and REM sleep. Raw EMG signals were full-wave rectified, integrated, and quantified in arbitrary units. Active wake was classified as low-amplitude EEG with high EMG activity. NREM sleep was classified as high-amplitude EEG dominated by delta band components (0–4 Hz). REM sleep was classified as low-amplitude EEG with low EMG activity. In addition to total sleep/wake time, power band (i.e., delta, theta, alpha, and beta) and power spectral (frequency) analysis of sleep/wake states was further assessed to study the quality of NREM and REM sleep in each animal.

### Immunohistochemistry

At the indicated times following rmTBI, the mice underwent transcardial perfusion with ice-cold 0.01 M phosphate-buffered saline (PBS) (pH 7.4), followed by fixation with 4% para-formaldehyde (PFA) in PBS. Brain tissue from all animals was dissected and post-fixed in 4% PFA for 24 h. Following fixation, the tissue underwent dehydration first in 30% sucrose for 24 h each. Tissue was placed in optimal cutting temperature (OCT) compound (Tissue-Tek) and was sliced on a cryostat (Microm HE 505E) into 30 μm coronal sections. Tissue sections were then floated in PBS. Six mice were included in each of the above groups. For each mouse, five representative coronal sections were selected for staining by collecting a single section every 1000 μm along a rostral–caudal axis beginning 1.1 mm anterior to and ending 2.5 mm posterior to bregma. The primary antibodies used included rabbit anti-mouse glial fibrillary acidic protein (GFAP) polyclonal IgG (Millipore, Billerica, MA, USA), mouse anti-human phospho-PHF-tau (pTau) monoclonal IgG (AT8, specific for pSer202/pThr205 tau phosphorylation sites) (Thermo Scientific, Rochester, Illinois, USA), and rabbit anti-mouse iba-1 (DAKO, Santa Clara, CA, USA) diluted to 1:1000. The secondary antibodies used were all diluted to 1:20,000 and included donkey anti-rabbit (Jackson ImmunoResearch, West Grove, PA, USA). All sections were blocked in 0.01 M PBS (pH 7.4) and 7% normal donkey serum [NDS] (VectorLabs, Burlingame, CA). Primary and secondary agents were diluted in 0.1% Triton X-100/PBS and 1% normal donkey serum.

### Image quantification

Immunohistochemical images were collected on a Nikon microscope, and image analyses were performed blinded to the experimental group. ImageJ software (http://rsbweb.nih.gov/ij/) was used to apply a standard threshold to the images.

### Western blot analyses

Relative levels of tau, p-tau, GFAP, iba-1, cathepsin B, and actin in the supernatant fraction from the brain extract were determined by Western blot (polyclonal antibodies: Cathepsin B, sc-13985; β-actin, sc-130657; Santa Cruz Biotechnology, Santa Cruz, CA; tau, ThermoFisher, Rochester, IL; p-tau, ThermoFisher, Rochester, IL; iba-1, DAKO, Santa Clara, CA), as described previously [34]. Relative intensities of Western blot bands were assessed by densitometry in triplicate for each sample. Densitometric analysis was done using IQTL software (GE Life Sciences, Piscataway, NJ). For protein studies, the entire lesional area was harvested for Western blot analysis. In control or sham animals, a similar region was harvested.

### ELISA analysis

For quantitative analysis of cytokines, an ELISA was used to measure the levels of tumor necrosis factor-α (TNF-α), interleukin-1β (IL-1β), or transforming growth factor-β (TGF-β) in the brain tissue [35]. Cytokines were extracted from mouse brains as follows: frozen hemibrains were placed in tissue homogenization buffer containing protease inhibitor cocktail (Sigma, St Louis, MO, USA) 1:1000 dilution immediately before use and homogenized using polytron. Tissue sample suspensions were distributed in aliquots and snap frozen in liquid nitrogen for later measurements. Invitrogen ELISA kits were then used, according to manufacturer directions (Carlsbad, CA, USA).

### Statistical analysis

All statistical analyses were performed using SAS statistical software version 9.3. All tests were two-sided and conducted at 5% significance level. Continuous variables were summarized using sample means. All studies used 10 mice per group. All data are presented as means ± standard error of the mean (SEM). Ipsilateral and contralateral sides were compared to the corresponding sides between groups [i.e., repetitive ipsilateral vs. single ipsilateral vs. control ipsilateral (left side)]. Normalized GFAP, pTau, and iba-1 immunoreactive areas were evaluated with thresholded pixel areas analyzed using one-way analysis of variance (ANOVA) including injury group (control, single hit, and repeated hits) as the factor. Post hoc analyses based on Tukey’s method to adjust for multiple comparisons were conducted to compare pairs of injury groups.

## Results

### Quantification of and immunolocalization of tau

In order to determine the impact of the nutritional diets on the development and progression of CTE, 24-week-old hTau mice were fed diets supplemented with GrandFusion diets (2%) for 2 months. The diets were as follows: group 3 received a 2% GrandFusion (GF1, NF-216—Fruit and Veggie #1 Blend), with the ND; group 4 received a 2% GrandFusion diet (GF2, NF-316—Fruit #2 Blend); and group 5 received a 2% GrandFusion diet (GF3, NF-416—Vegetable #3 Blend) (Table 1). The diets contain similar level of vitamins, phytochemicals, and phytonutrients that might impact the outcomes. These are same diets that were used in previous studies [27]. The animals were examined for food intake and body weight every week for the 24 weeks of feeding. The mice on all diets maintained a constant intake of food over the course of the study (data not shown). In addition, consistent with the food intake, all of the mice showed a similar gain in weight over the 8 months.

**Fig. 1:**
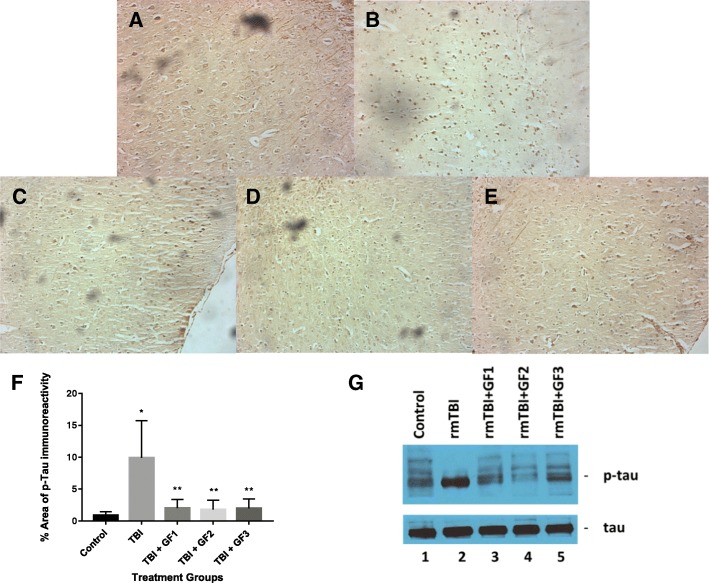
Effects of GF diets on tau pathology. Control hTau mice (**a**), hTau mice + rmTBI (**b**), hTau mice + rmTBI plus NF-216 (**c**, GF1), hTau mice + rmTBI plus NF-316 (**d**, GF2), and hTau mice + rmTBI plus NF-416 (**e**, GF3). Mice were fed a normal diet or diets supplemented with 2% GF for 2 months prior to rmTBI and then for 4 months after rmTBI. Animals were euthanized and subjected to immunohistochemical analysis (**a**–**f**) or Western blot analysis (**g**). **f** Graphical representation of p-tau immunohistochemistry in **a**–**e**. Each group represents mean ± SD (*n* = 10 per group). *p < 0.001 compared to TBI group

The mice were subjected to closed head rmTBI as described previously [28]. Mice were examined for phosphor-tau (p-tau) presence in the brain following rmTBI and the impact of the diets on altering p-tau expression. Figure 1 shows that control mice at 14 months of age show little p-tau pathology (Fig. 1a). Mice subjected to rmTBI showed a dramatic increase in p-tau pathology compared to the control animals (Fig. 1b). With the presence of the diets, there was a significant reduction in the p-tau pathology suggesting the diets had an effect on rmTBI-induced outcomes (Fig. 1c–e). Western blot analysis of the mice from the above studies shows the changes in p-tau versus tau in the brains of the mice with and without rmTBI and with and without GF diets (Fig. 1f, g). As seen in the figure, with rmTBI, the levels of p-tau are increased 5–10 fold compared to the control animals, while the mice on the GF diets showed an attenuation of p-tau expression.

### RmTBI results in a transient Neurological Severity Score elevation and short-lived motor deficits that are ameliorated with diets

**Fig. 2:**
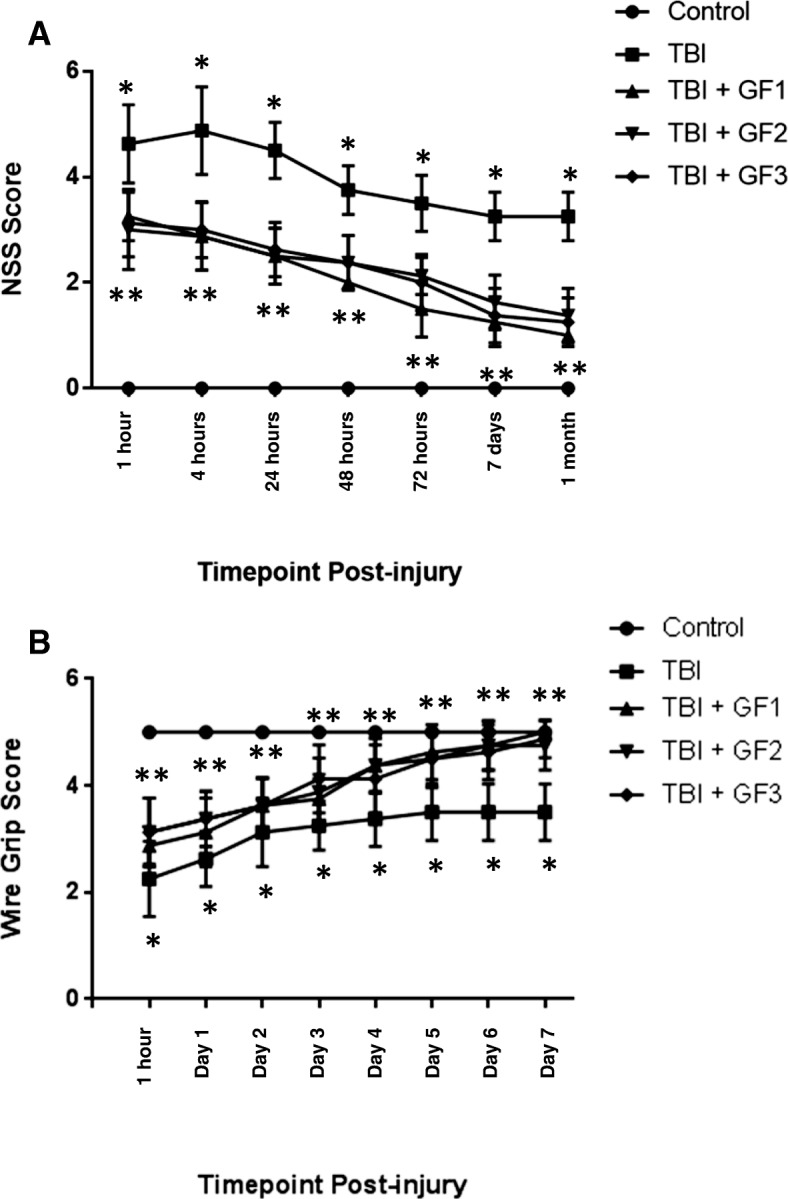
Repetitive mild traumatic brain injury (rmTBI) results in elevated Neurological Severity Scores (NSS) and transient vestibulomotor deficits. **a** rmTBI mice +/− were assessed with an NSS at 1, 4, 24, 48, and 72 h post-injury, as well as at 7-day and 1-month time points. Repetitive mTBI mice exhibited significantly elevated scores, compared to control mice, and mice fed GF diets had reduced NSS score compared to rmTBI alone. **b** Mice underwent wire grip testing 1 h after TBI and on post-injury days 1–7. rmTBI resulted in short-lived vestibulomotor dysfunction, compared to controls, at 1 h to 7 days post-injury (Kruskal-Wallis). There were significant differences between rmTBI mice plus diets and rmTBI alone on post-injury days 1–7. *p < 0.05 versus control mice. **p < 0.01 versus rmTBI mice. Values are mean ± SD

In NSS testing, severity of impact for rmTBI groups fell in the mild spectrum. We found that averaged NSS scores were statistically different between the injury and treated groups and that the effect of the diets on outcomes was significant. Figure 2a shows that rmTBI at 6 months post-injury, the NSS was significantly higher in the rmTBI group compared to the control group. In addition, the animals on the diets had an attenuation of the NSS following rmTBI.

Vestibulomotor function was assessed by WGT, and there were significant effects of injury group on performance. We found that averaged wire grip scores were statistically different between rmTBI group and the control group and that the effect of injury was significant at 1 h to day 7 post-injury, even after adjustment for multiple comparisons (*p*_adj_ < 0.05; Kruskal-Wallis). Post hoc analyses found significant difference at the 5% significance level, at 1 h to 7 days post-injury, with the rmTBI group statistically different from the control group. In addition, the GF groups all showed a significant difference compared to the rmTBI group. We also found that the performance on the WGT improved over time, with wire grip scores increasing over time in the repeat injury group (both groups, *p* < 0.001; Friedman).

### Impact of rmTBI and diets on cytokine levels

**Fig. 3:**
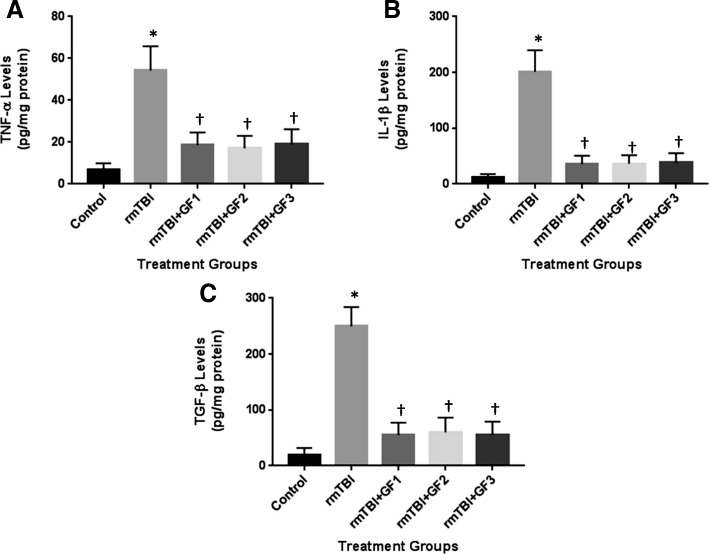
Reduced inflammatory markers in the brain after rmTBI. Mice were grouped as control, rmTBI, or rmTBI subjected to various diets followed by 24 h of recovery. Quantitative analysis of TNF-α (**a**), IL-1β (**b**), and TGF-β (**c**) in the rmTBI brain was determined by ELISA. Brain homogenates were subjected to ELISA. The results are expressed as mean ± SD (*n* = 10, *P < 0.001 compared to the sham group; < 0.001 compared to the rmTBI group)

To determine the impact of the diets on neuroinflammation in the mouse brain after rmTBI, mouse brains were examined for the expression of inflammatory markers. We evaluated the levels of the cytokines tumor necrosis factor-α (TNF-α), interleukin-1β (IL-1β), and transforming growth factor-β (TGF-β) at 6 months after rmTBI (Fig. 3). As seen in the figure, rmTBI that resulted in “CTE-like” effects elevated cytokine levels that were still increased at 6 months after injury. The GF diets significantly reduced or attenuated TNF-α, IL-1β, and TGF-β levels after injury. All the diets showed an effect reducing the above cytokine levels by 67% (TNF-α), 85% (IL-1β), and 80% (TGF-β).

### Changes in cathepsin B levels following rmTBI and effect of diets

**Fig. 4:**
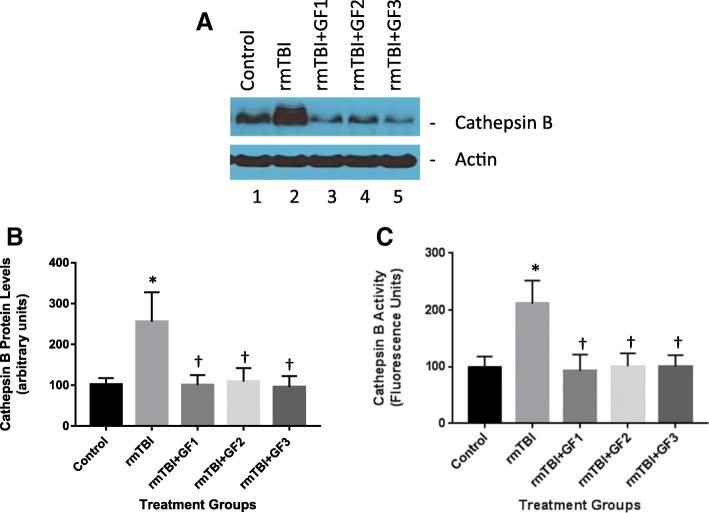
The effect of GF diets on cathepsin protein levels and B activity. **a** Brain cathepsin B protein levels were determined 4 months following rmTBI. Western blot analysis of the cathepsin B levels in the brains of control, rmTBI, and rmTBI + GF diets. **b** Quantitative analysis of cathepsin B protein levels of the mice in **a**. **c** Brain cathepsin B activities were determined in the mice following 4 months after rmTBI in control, rmTBI, and rmTBI + GF diets. The results are expressed as mean ± SD (*n* = 10, *p < 0.001 compared to the control group; †*p* < 0.01 versus rmTBI group)

Our previous studies have shown that TBI results in an increase in cathepsin B protein and activity that can lead to inflammatory mediators such as IL-1β. To determine the impact of rmTBI on cathepsin B levels and diets associated with the alterations in inflammation (Fig. 4), we measured cathepsin B protein and activity at 4 months following injury. rmTBI increased cathepsin B levels in the brain, and the GF diets reduced or attenuated the increase (Fig. 4). These results suggest that reduction in inflammation occurring with treatments was partially the result of inhibition of cathepsin B activity.

### Impact of rmTBI and diets on glial activation following rmTBI

**Fig. 5:**
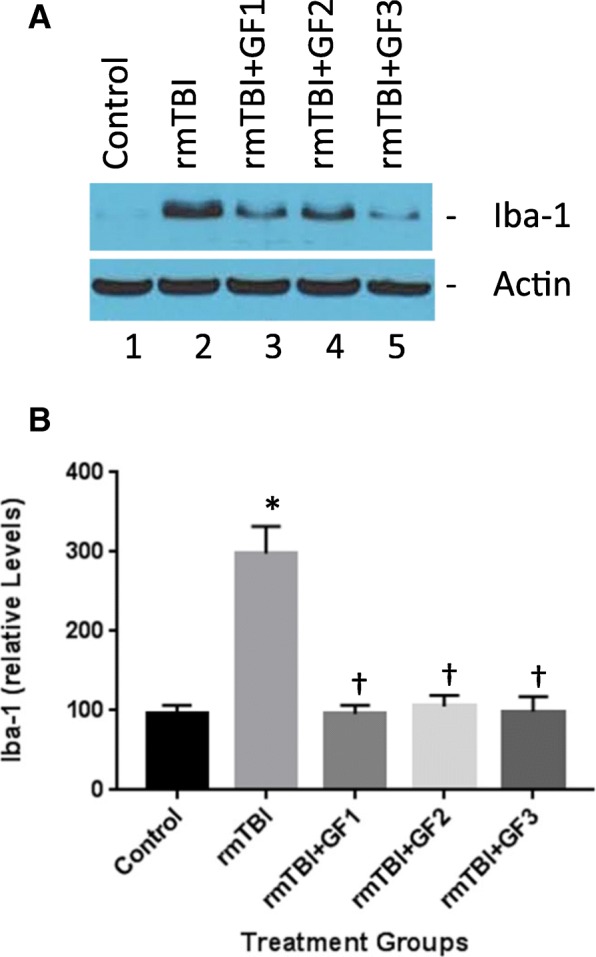
Reduced inflammatory markers in the brain after rmTBI. Mice were grouped as control, rmTBI, or rmTBI subjected to various diets followed by 4 months of recovery. **a** Western blot analysis of iba-1 (activated microglia) was determined in the mice. **b** Quantitative assessment of the Western blot in **a**. The results are expressed as mean ± SD (*n* = 10, *p < 0.001 compared to the control group; †*p* < 0.001 compared to the rmTBI group)

**Fig. 6:**
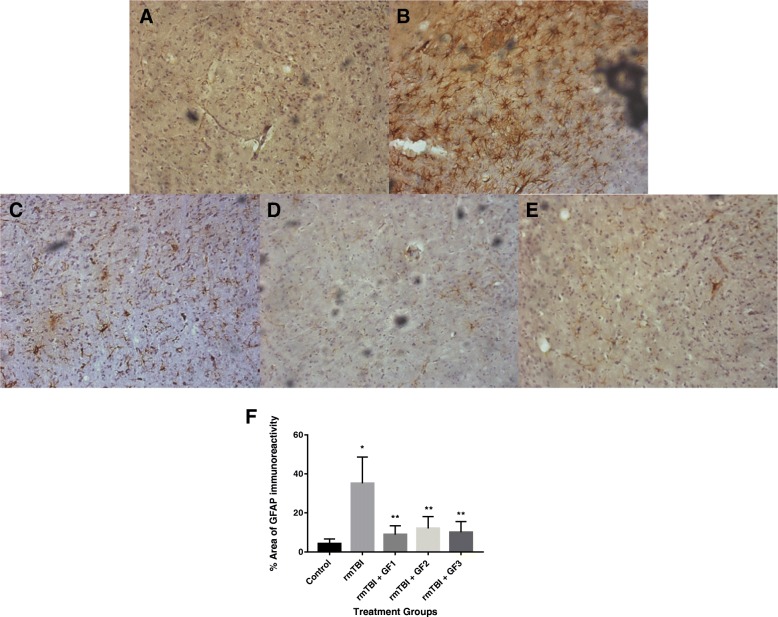
Reduced inflammatory markers in the brain after rmTBI. Mice were control, rmTBI, or rmTBI subjected to various diets followed by 4 months of recovery. **a**–**e** Immunohistochemical analysis of GFAP (activated astrocytes) was determined in the mice. Control hTau mice (**a**), hTau mice + rmTBI (**b**), hTau mice + rmTBI plus NF-216 (**c**, GF1), hTau mice + rmTBI plus NF-316 (**d**, GF2), and hTau mice + rmTBI plus NF-416 (**e**, GF3). **f** Quantitative assessment of the western histology in **a**–**e**. The results are expressed as mean ± SD (*n* = 10, *p < 0.001 compared to the control group; ** *p*< 0.001 compared to the rmTBI group)

To further analyze the impact of the diets on neuroinflammation in the mouse brain after rmTBI, brains were examined for the expression of glial inflammatory markers. We evaluated the levels of the astrocyte (GFAP) and microglial (iba-1) at 6 months after rmTBI (Figs. 5 and 6). As seen in the figures, rmTBI that resulted in “CTE-like” effects elevated both iba-1 (Fig. 5) and GFAP (Fig. 6) levels that were still increased at 6 months after injury. The GF diets significantly reduced or attenuated iba-1 and GFAP levels after injury. All the diets showed an effect reducing the above glial markers by 67% (iba-1) and 82% (GFAP).

### Repetitive mild traumatic brain injury causes persistent deficits with spatial learning and memory

**Fig. 7:**
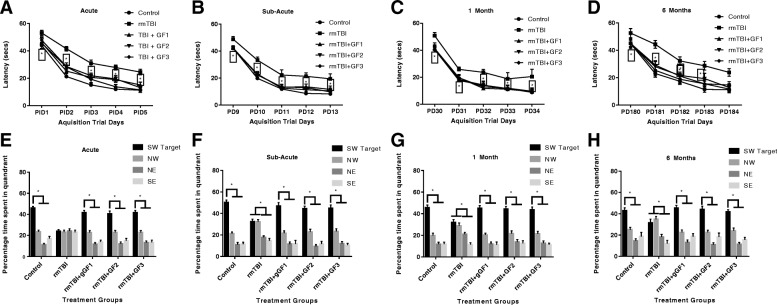
Evaluation of learning (acquisition) and spatial memory retention (probe) using the Morris water maze. **a**–**d** During acquisition training sessions, repetitive mTBI mice demonstrated a persistent, significant increase in escape latency acutely, subacutely, and at 1- and 6-month post-injury (*p* < 0.05 at all time points; Tukey). All treatments with GF diets returned the levels to normal. **e**–**h** During probe trials, control and single mTBI mice demonstrated spatial memory retention, spending a significantly greater percentage of time in the target quadrant (SW), compared with all other quadrants (*Min* test). At the acute time point, rmTBI mice spent a similar percentage of time in all quadrants, no greater than chance, and also did not show a preference for the target quadrant, compared with the other quadrants subacutely and at 1 and 6 months post-injury (*Min* test). All treatments with GF diets returned the levels to normal. *p < 0.05. Values are mean ± SD. NW, northwest; NE, northeast; SE, southeast; SW, southwest

We next determined the hippocampal-dependent spatial learning and long-term memory in the rmTBI and rmTBI + GF diets mice using the MWM (Fig. 7). Animals from all groups showed daily improvements in their abilities to locate the hidden platform during the acquisition phase of the MWM task; however, rmTBI mice demonstrated increased latencies. Mice maintained on the DF diets showed an attenuation of the changes and were similar to the control animals. At the acute and subacute time points, we found that the main effect of time, the main effect of injury group, and the interaction of time and injury group were statistically significant (Fig. 7a, b). At 1 month post-injury, the main effect of time and the main effect of injury group were statistically significant, and the GF diets were significantly different compared to the rmTBI group (Fig. 7c). At 6 months post-injury, we found that time and the interaction of time and injury group were significant and that the main effect of GF injury group was significant to the rmTBI group (Fig. 7d). Post hoc analyses found that, at the acute time point, all pairs of injury groups (control vs. repetitive mTBI) were statistically different at the 5% significance level. At the subacute time point, the analyses found statistically significant differences between the control and the repetitive mTBI groups. At 1 month post-injury, we found statistically significant differences when comparing control to repetitive mTBI groups. At 6 months, we found statistically significant differences when comparing control to repetitive mTBI groups. In addition, the diet-treated animals showed significant difference at all time points compared to the rmTBI group.

For the probe trial testing, analyses comparing the control groups found significant differences in the distribution pattern of the time that mice spent in the four quadrants at any of the time points. When comparing the rmTBI and the control injury group, we found significant differences in the distribution of the time that mice spent in the four quadrants. When analyzing within each time point, we found significant differences between the control and the repeat group at the acute time point. The difference was significant at both the subacute and 1-month time points and at 6 months. In addition, the diet-treated animals showed significant difference at all time points compared to the rmTBI group.

Subsequent analysis was performed to evaluate the preference for the target quadrant compared to the other three quadrants. Findings from the probe test indicate that mice from the uninjured control groups, at all time points, spent a significantly higher percentage of time in the target quadrant (the location that contained the platform during training), when compared to the other equivalent zones (Fig. 7e–h). We found that mice in the control group spent significantly more time in the target quadrant than in any of the other three quadrants at the acute, subacute, 1-month, and 6-month time points post-injury. In contrast, rmTBI mice exhibited impaired spatial memory, failing to show significant discrimination and preference for the target quadrant, compared to the other quadrants (Fig. 7e–h). We did not find that mice in the repetitive injury group spent significantly more time in the target quadrant than in any other quadrants at any of the post-injury times. Meanwhile, mice fed GF diets showed a significant increase in the time spent in the target quadrant and less time in any other quadrant (Fig. 7e–h).

### rmTBI resulted in subacute anxiety leading to increased risk-taking activity which is attenuated in GF diets

**Fig. 8:**
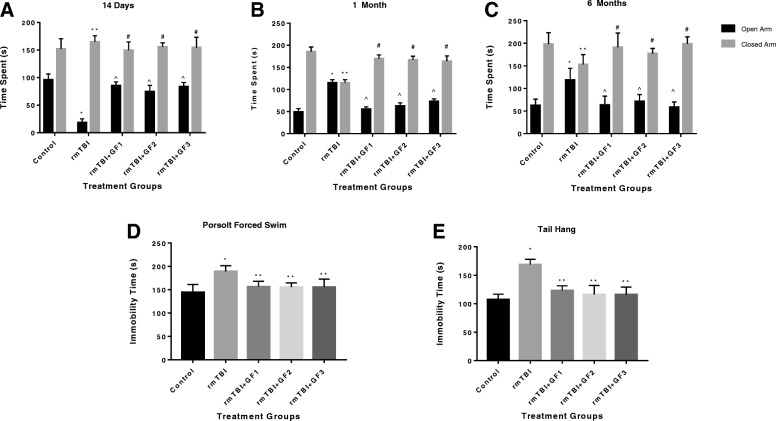
Repetitive mild traumatic brain injury (rmTBI) results in increased risk-taking and depression-like behaviors. **a** rmTBI results in reduced time spent on the open arms of the elevated plus maze (EPM), consistent with increased anxiety at 2 weeks post-injury (Tukey). All treatments with GF diets returned the levels to normal. **b** At 1 month post-injury, repetitive mTBI mice spend more time on the open arms of the EPM, compared to control mice, consistent with decreased fear avoidance and increased risk taking (Tukey). All treatments with GF diets returned the levels to normal. **c** By 6 months post-injury, risk-taking behavior progressively increases in the repetitive mTBI mice (Tukey). All treatments with GF diets returned the levels to normal (**d**, **e**). Repetitive mTBI mice also demonstrated increased immobility time in the Porsolt forced swim and tail suspension tests, consistent with depression-like behavior at 1 month post-injury (Tukey). All treatments with GF diets returned the levels to normal. *p < 0.05 versus control. Values are mean ± SD

We used the EPM to determine the effect of rmTBI on anxiety-related and risk-taking behaviors. One-way ANOVA revealed that the amount of time spent in the open arm differed significantly between the control and injury group +/− diets at 14 days, 1 month, and 6 months. At 2 weeks post-injury, mTBI mice exhibited increased anxiety-like behavior (Fig. 8a). Post hoc analyses found significant differences at the 5% significance level at 14 days post-injury between the control and the repetitive injury groups. rmTBI resulted in significantly reduced time spent in the open arms of the maze, consistent with increased anxiety. At day 14 post-injury, the mice with GF diets showed an increased time in the open arms consistent with decreased anxiety.

At the 1- and 6-month time points, significant differences were found in post hoc analyses between the controls and repetitive injury group. Mice in the rmTBI group spent an increased amount of time on the open arms of the EPM, compared to control mice (Fig. 8b). Such increased exploratory activity in the open arms and reduced fearfulness are consistent with increased risk taking, as noted in other studies [36]. This increased risk taking persisted and progressed in the rmTBI mice out to 6 months (Fig. 8c). However, the GF diet-fed mice showed an attenuation of the presence in the open arm supports decreased risk-taking and a maintenance of normal activity.

### Repetitive mild traumatic brain injury results in depression-like behavior at 1 month

At 1 month post-injury, in the Porsolt FST, there was a significant effect of injury severity on depression-like behavior. Data from the swim test indicated significant differences between groups. Post hoc analyses found significantly increased immobility time in the rmTBI group, compared to the control group (Fig. 8d). The TST revealed comparable effects of mTBI on depression-like behavior as the FST. Similar analyses carried out on data from the TST suggested that immobility times differed significantly between injury groups. rmTBI mice demonstrated significantly increased immobility time, compared to the control group (Fig. 8e). The rmTBI mice on GF diets showed a return to control levels in both the FST and TST suggesting protective effects on the rmTBI. No differences were seen in the TST (data not shown).

### Mild traumatic brain injury mice exhibit sleep disturbances at 1 month

**Fig. 9:**
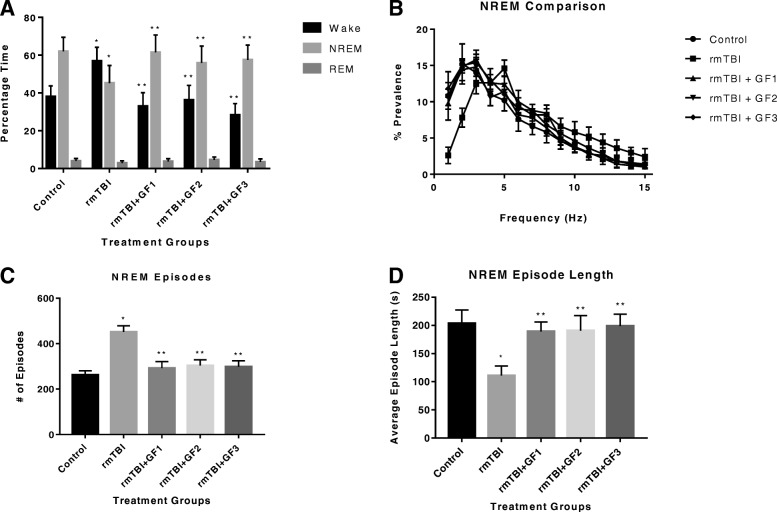
Repetitive mild traumatic brain injury (rmTBI) results in sleep pattern disturbances (**a**). Mice repetitive rmTBI exhibit a significant reduction in NREM sleep as well as a significant increase in wake time over the course of 24 h (Tukey). *p < 0.05, compared to control mice. **b** Quality of NREM sleep is disrupted by rmTBI. Power spectral analysis demonstrated a significant rightward theta shift in rmTBI animals. Control animals displayed a significantly higher power spectrum for 1–2 Hz, compared to rmTBI groups. Single and repetitive rmTBI mice had a significantly higher frequency at 5 Hz, compared to age-matched controls (Tukey). Single rmTBI mice also had a significant difference at 12 and 13 Hz, compared to control mice (Tukey). *p < 0.05, repetitive rmTBI versus control mice; ^#^*p* < 0.05, single rmTBI versus control mice. **c** Repetitive rmTBI mice exhibited a greater number of NREM episodes, compared to the single rmTBI and control groups, with **d** significantly reduced episode lengths (Tukey). *p < 0.05. Values are mean ± SD. NREM, non-rapid eye movement; REM, rapid eye movement

To evaluate the long-term effect of rmTBI +/− diets on sleep-wake behavior, we used infrared videography and electrophysiological monitoring [37]. We determined that the effect of rmTBI on percent wake time was statistically significant (Fig. 9a). The percentage of wake time in the rmTBI group was significantly different from those recorded in the control group. With the significant increase in wake time, we found a concomitant reduction in NREM sleep in the rmTBI mice. We found that the effect of injury group on percent NREM time was statistically significant. The percentage of NREM time in the rmTBI group was significantly different from those recorded in the control group. We also found that the effect of the rmTBI on the percent REM time was not statistically significant. However, mice on the diets showed a decrease in wake time and an increase in NREM time (statistically significant).

We sought to further examine the quality of NREM and REM sleep in these mice. During NREM sleep, there was an increase in cortical activity with a significant shift toward higher frequencies (Fig. 9b). The prevalence was significantly different across levels of frequency, and the rmTBI group had a significant effect on prevalence through its interaction with frequency. Analyses found that the effect of rmTBI group on frequency prevalence was statistically significant at all frequency levels compared to the control group. In addition, we found significant difference between the rmTBI and the rmTBI + GF diets, suggesting that there was an impact by the diets on the NREM activity.

**Fig. 10:**
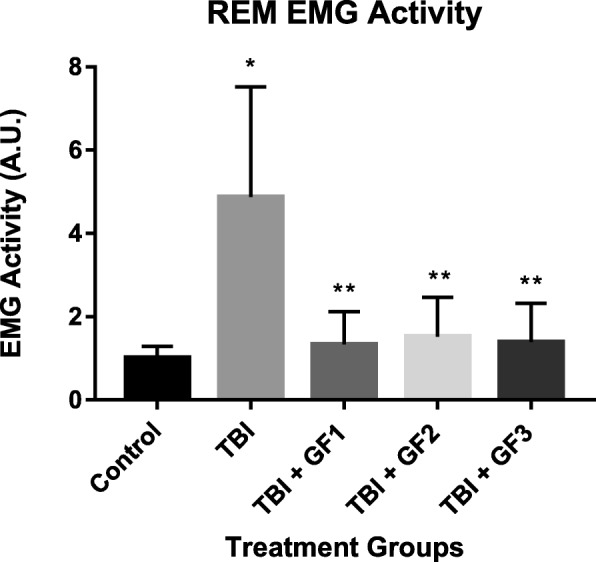
Repetitive mild traumatic brain injury (rmTBI) results in abnormal REM EMG. Repetitive mTBI mice demonstrated significantly higher EMG activity than controls during REM sleep (Tukey). All treatments with GF diets returned the levels to normal. *p < 0.05, compared to control mice; **p < 0.05, compared to rmTBI mice. Values are mean ± SD. REM, rapid eye movement; EMG, electromyography; A.U., arbitrary units

Repetitive mTBI also caused NREM sleep fragmentation as well (Fig. 9c, d). We found that the rmTBI group had a significant effect on both the number of episodes and average episode length. The number of episodes in the rmTBI mice was significantly increased from those observed in the control group (Fig. 9c). The average episode length in the rmTBI mice was also significantly reduced, compared to control mice (Fig. 9d). Meanwhile, mice on the GF diets subjected to rmTBI had attenuated NREM episodes and increased NREM episode length, comparable to the control animals. Both were statistically significant compared to the rmTBI animals. Effect of injury was significant on REM EMG data. Analyses revealed that the rmTBI mice demonstrated significantly increased EMG activity, compared to controls, during REM sleep (Fig. 10). However, mice on the GF diets showed an attenuation of the REM EMG activity.

## Discussion

In the present study, we examined the impact of diets rich in vegetables and/or fruits on outcomes and recovery/repair from rmTBI and the potential link to CTE. Our studies have shown that long-term intake of these diets for 2 months prior to rmTBI and 6 months subsequent to the injury improved behavioral outcomes, reduced inflammation, and diminished tauopathy in a mouse model of CTE.

A number of recent studies have implicated rTBI in the pathogenesis associated with CTE [38]. A chronic increase in phosphorylated tau (p-tau) immunostaining has been detected in the cortex, amygdalae, and the hippocampus of individuals subjected to rTBI. A number of models have been developed to study the mechanisms associated with rTBI and CTE [39]. For the lack of a better model, a rmTBI seems to be the most relevant approach to study the pathophysiology related to CTE [40]. In these models, p-tau staining was associated with increased GFAP-positive astrocytes and iba-1-positive microglial cells [41]. These markers are consistent with the appearance in reported cases of CTE and individuals that experience chronic mild repetitive head traumas [42]. The presence of the inflammation and glial markers is most likely due to several different parameters [43]. The microglial activation and reactive astrocytosis are probably the result of the primary injury, the repetitive mTBI, and the consequences of a progressive, chronic neuroinflammatory condition that contributes to secondary and potentially tertiary responses [44]. In addition, the presence of the p-tau and deposition may contribute to the continued inflammation, i.e., inflammation begets inflammation [45]. The resulting injury and inflammation contributes to glial activation and neuronal cell death that will give rise to more inflammation and glial activation and more cell death, etc. They may be one of the main issues related to CTE [46]. We continue to see inflammation at the 6-month time point in our model suggesting that chronic inflammation is critical to the disease process [47].

Recent studies from our group have demonstrated that application of nutrient rich diets may alter the outcomes associated with neurological disorders, aging, and TBI [27, 34]. As shown previously, mice provided a diet enriched in fruits and vegetables help to attenuate the damage instigated by middle cerebral artery occlusion (MCAo) and maintain behavioral parameters [27]. These studies also further validated that phyto-nutriceuticals were capable of limiting inflammation and oxidative stress while stimulating neuronal proliferation. We also showed that when aged rats were provided the GF diets, there appeared to be an effect upon the aging process by a reduction in inflammatory markers, oxidative stress, and an increase in behavioral movement [34]. A recent related study showed that when mice were pre-exposed to these diets, there was a protection from the detrimental effects of TBI. When exposed to controlled cortical impact, the mice showed cortical damage, increased inflammation, and behavioral deficits. However, when exposed to GF diets, the mice had preserved neuronal function, reduced inflammatory markers, and improved or attenuated outcomes. As seen in the behavioral studies, most of the behavioral outcomes were suppressed but not completely obviated following TBI as seen in other studies, while the grip-strength showed a complete recovery. The grip strength test is not as selective as some of the other test; therefore, it needs to be taken within the complete context of the study. These data suggest that consumption of diets enriched in fruits and vegetables either naturally or through powdered form can provide protection from the detrimental effects of injury.

We describe the impact of long-term treatment of mice to diets enriched with vegetable and/or fruit concentrates in a model of repetitive mild TBI. Mice subjected to rmTBI showed chronic inflammatory responses and increased tau phosphorylation out to 6 months. The neuroinflammatory response with GFAP and iba-1 persisted out to the 6-month time point. The application of the GF diets to the mice, pre- and post-injury, reduced the neuroinflammation as apparent with both cytokine and glia activation. In addition, behavior and p-tau were both attenuated in the animal model suggesting an impact on the disease process. The studies further define the interplay between neuroinflammation and tau phosphorylation or vice versa, in the pathology and behavioral manifestations. They demonstrated that diets containing anti-oxidants, anti-inflammatory agents, and other compounds may have some interventional aspect in a mouse model of “CTE” and may provide a potential preventative/therapeutic approach. While in the context of a “real world” setting, predicting when and where TBI(s) might occur is not possible to preload with phytochemicals and phytonutrients. However, the studies suggest that using a diet like used in the study will help to attenuate the damage caused by TBIs.

## Conclusion

Here, we show that treatment of mice with diets enriched in fruits and vegetables (phytochemicals) can alter the pathogenesis of CTE. Although treatment was started prior to the CTE, the indications are that the presence of these diets helped to attenuate the disease process, reduce inflammation, and improve outcomes. These data suggest that diets enriched in phytochemicals and other entities will help to limit the extent of injury following TBIs and reduce the potential progression to CTE in individuals.

## Abbreviations

CTE: Chronic traumatic encephalopathy
GFAP: Glial fibrillary acidic protein
IL-1β: Interleukin-1beta
TBI: Traumatic brain injury
